# Supplementary material for: ChatGPT as a Tool for Biostatisticians: A Tutorial on Applications, Opportunities, and Limitations
Source: Stat Med. 2025 Oct 23;44(23-24):e70263. doi: 10.1002/sim.70263 (PMC12548020; doi:10.1002/sim.70263)
Supplement: Supplementary file 1 — Data S1. Supporting Information. [file SIM-44-0-s001.zip › Supplementary Materials Additional.zip!/3.1_Systematic_Reviews_and_Meta_aspects/3.1_Forest Plot - 4o - Chat 7.pdf]

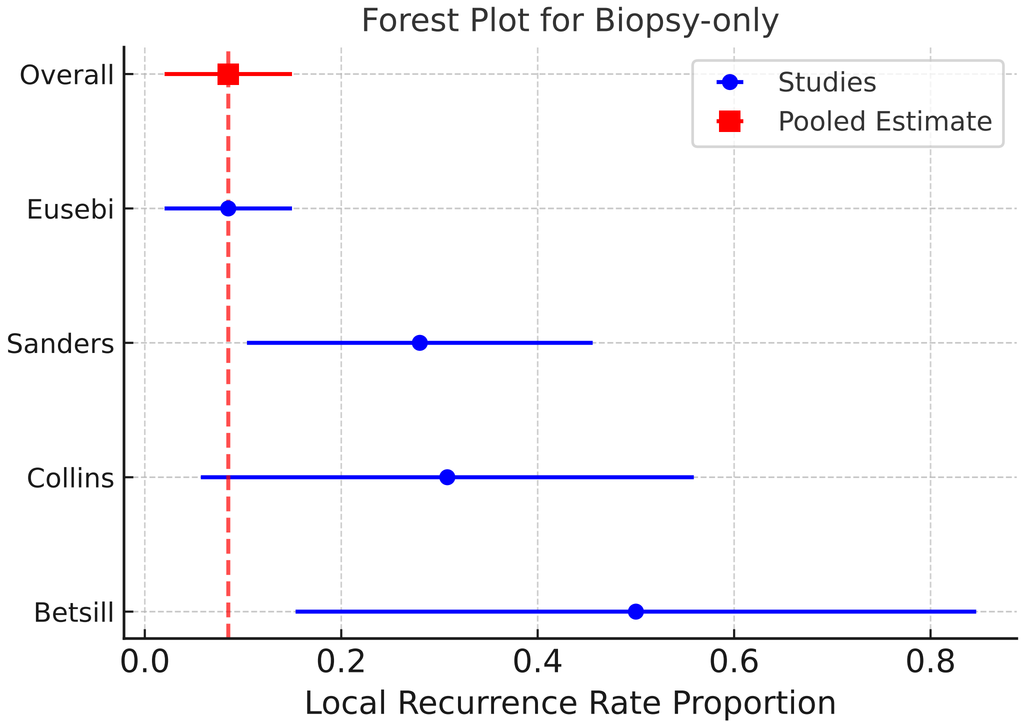
Forest Plot for Biopsy-only
Studies
Pooled Estimate
Overall
Eusebi
Sanders
Collins
Betsill
0.0
0.2
0.4
0.6
0.8
Local Recurrence Rate Proportion

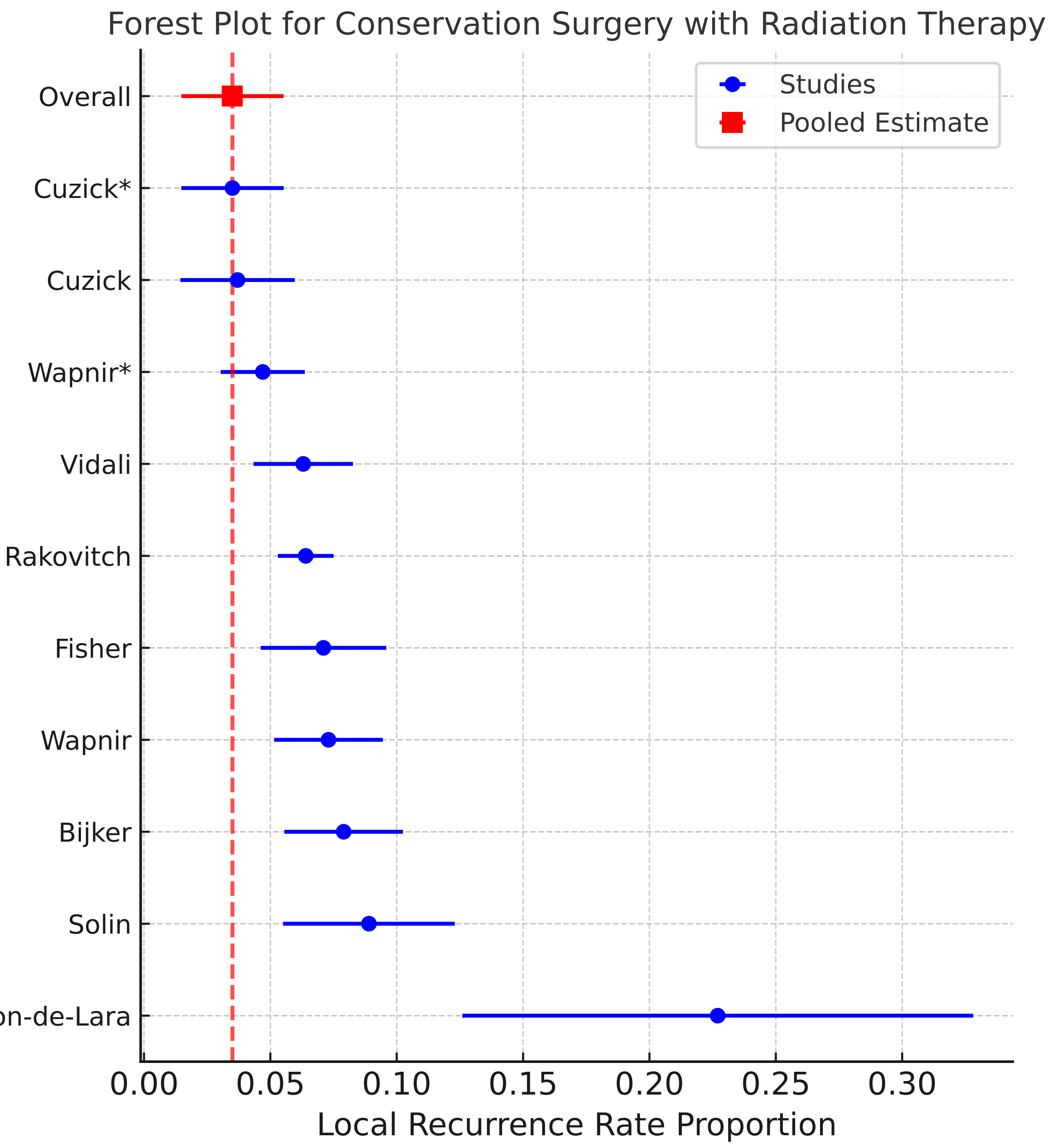

Forest Plot for Conservation Surgery with Radiation Therapy
Studies
Pooled Estimate
Overall
Cuzick*
Cuzick
Wapnir*
Vidali
Rakovitch
Fisher
Wapnir
Bijker
Solin
on-de-Lara
0.00
0.05
0.10
0.15
0.20
0.25
0.30
Local Recurrence Rate Proportion

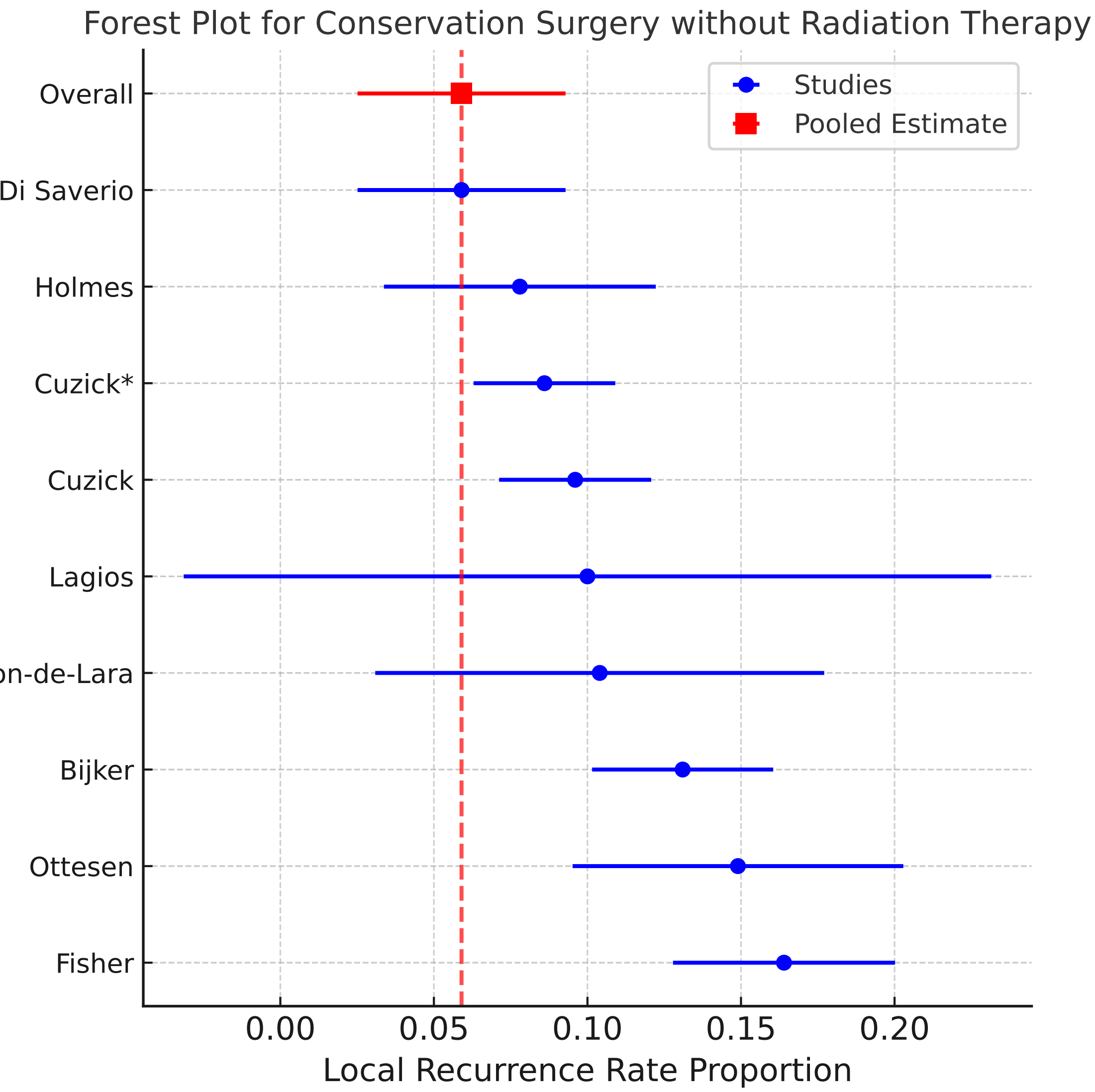

Forest Plot for Conservation Surgery without Radiation Therapy
Studies
Pooled Estimate
Overall
Di Saverio
Holmes
Cuzick*
Cuzick
Lagios
on-de-Lara
Bijker
Ottesen
Fisher
0.00
0.05
0.10
0.15
0.20
Local Recurrence Rate Proportion

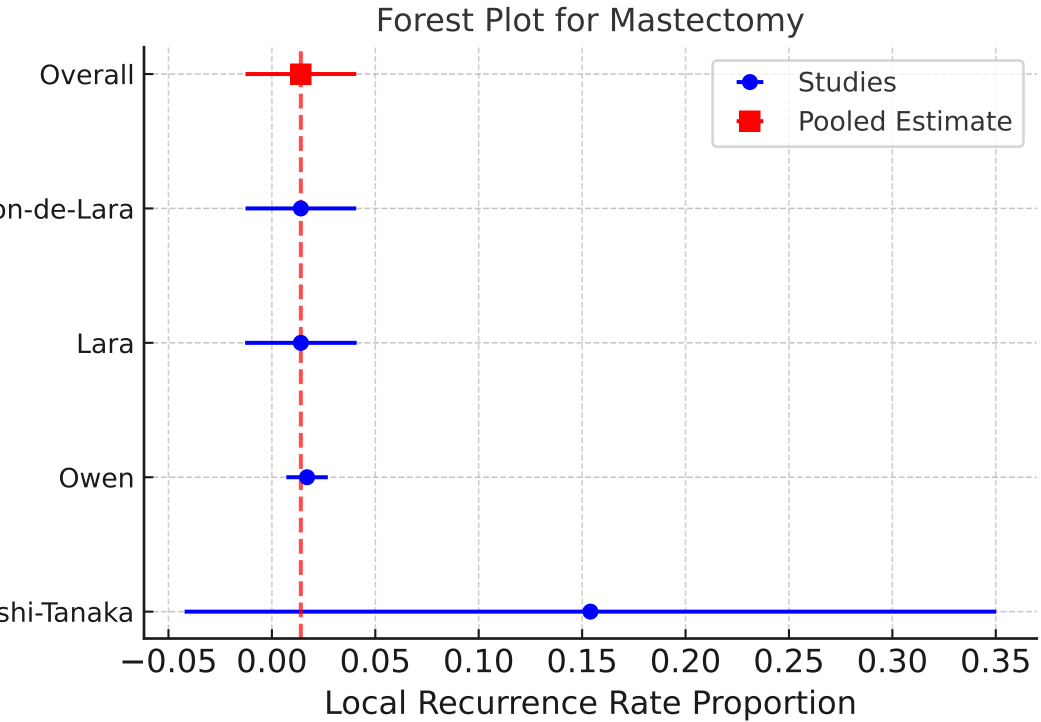
Forest Plot for Mastectomy
Overall
on-de-Lara
Lara
Owen
shi-Tanaka
Studies
Pooled Estimate
−0.05
0.00
0.05
0.10
0.15
0.20
0.25
0.30
0.35
Local Recurrence Rate Proportion
